# Triptycene-like naphthopleiadene as a readily accessible scaffold for supramolecular and materials chemistry†

**DOI:** 10.1039/d4sc02755h

**Published:** 2024-08-27

**Authors:** Md Khairul Amin, Chunchun Ye, Shuhua Pang, Yuancheng Liu, Dominic Taylor, Gary S. Nichol, Neil B. McKeown

**Affiliations:** a EaStCHEM, School of Chemistry, University of Edinburgh David Brewster Road Edinburgh EH9 3FJ UK neil.mckeown@ed.ac.uk; b Chemistry Discipline, Khulna University Khulna 9208 Bangladesh

## Abstract

Triptycene derivatives are used extensively in supramolecular and materials chemistry, however, most are prepared using a multi-step synthesis involving the generation of a benzyne intermediate, which hinders production on a large scale. Inspired by the ease of the synthesis of resorcinarenes, we report the rapid and efficient preparation of triptycene-like 1,6,2′,7′-tetrahydroxynaphthopleiadene directly from 2,7-dihydroxynaphthalene and phthalaldehyde. Structural characterisation confirms the novel bridged bicyclic framework, within which the planes of the single benzene ring and two naphthalene units are fixed at an angle of ∼120° relative to each other. Other combinations of aromatic 1,2-dialdehydes and 2,7-disubstituted naphthalenes also provided similar triptycene-like products. The low cost of the precursors and undemanding reaction conditions allow for rapid multigram synthesis of 1,6,2′,7′-tetrahydroxynaphthopleiadene, which is shown to be a useful precursor for making the parent naphthopleiadene hydrocarbon. The great potential for the use of the naphthopleiadene scaffold in supramolecular and polymer chemistry is demonstrated by the preparation of a rigid novel cavitand, a microporous network polymer, and a solution-processable polymer of intrinsic microporosity.

## Abstract 2

The efficient one-step synthesis of the triptycene-like 1,6,2′,7′-tetrahydroxynaphthopleiadene provides a useful precursor for a novel cavitand, microporous polymers, and the parent naphthopleiadene.

## Graphical abstract

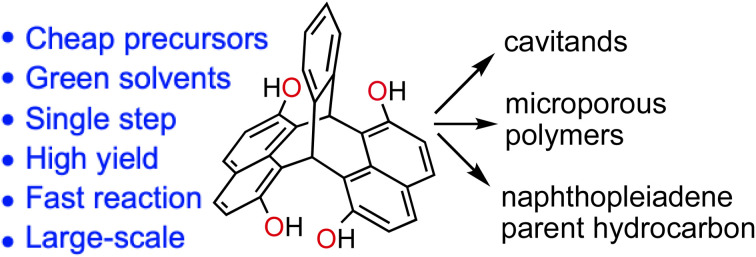

## Introduction

**Fig. 1:**
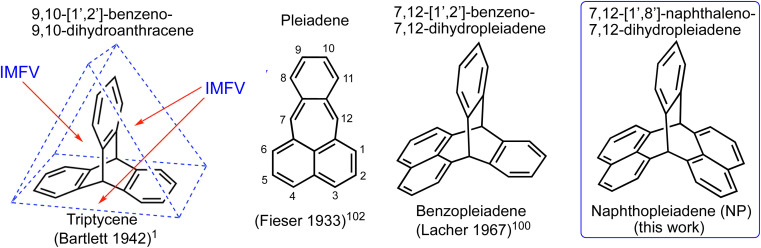
The structure and nomenclature of triptycene, pleiadene and the two triptycene-like molecular frameworks obtained from the formal fusing of either benzene or naphthalene, *via* two of its *peri*-carbons, to the 7,12 sites of pleiadene. The intramolecular free volume (IMFV)^73^ is indicated for triptycene and this will be enhanced for naphthopleiadene due to the greater width of the naphthalene units.

Triptycene is a bridged bicyclic hydrocarbon composed of three benzene rings fused to two sp^3^ hybridised carbon bridgeheads (Fig. 1).^1–3^ The symmetry and rigidity of triptycene results in its derivatives being used extensively as scaffolds in supramolecular^4–10^ and materials chemistry.^11–20^ For example, triptycenes are key components in molecular machines (such as gears,^21–24^ brakes,^25^ rotors^26^), crystal engineering,^27,28^ macrocycles,^7,29,30^ cages,^31–33^ optoelectronic materials,^34–40^ self-assembling monolayers at interfaces,^41–45^ and ligands for metal catalysts.^46–50^ The shape of triptycene, with the three benzene rings fused at an angle of 120° relative to each other, is particularly attractive for providing the vertices within ordered porous materials such as 2D honeycomb-like polymers,^51,52^ molecular crystals,^53,54^ nanotubes,^55^ covalent organic frameworks,^56–58^ and metal organic frameworks.^26,59^ However, our long-term interest in triptycene derivatives has been in their use as monomers for making amorphous Polymers of Intrinsic Microporosity (PIMs),^60–63^ an interest which is shared with several other research groups.^64–71^ PIMs exploit the rigidity^72^ of triptycene and the internal molecular free volume (IMFV)^73^ that originates from its concavities (Fig. 1). Triptycene-derived PIMs have great potential for making high performance gas separation membranes.^74,75^ Indeed, data from these polymers define the current upper bounds for the trade-off between permeability and selectivity for the separation of several important gas pairs,^76,77^ including those of interest for post combustion carbon capture and natural gas purification.^78^

**Fig. 2:**
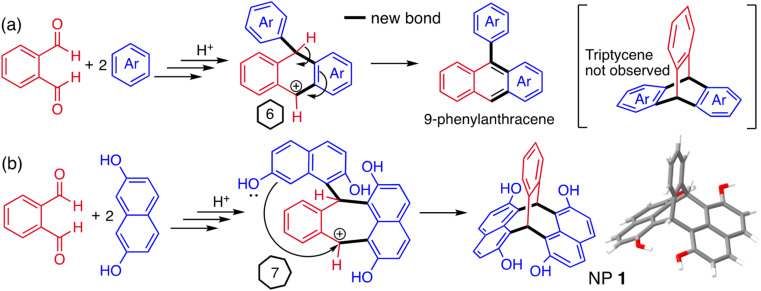
Proposed partial mechanisms for (a) formation of 9-phenylanthracenes from the reaction between phthalaldehyde and benzene derivatives (Ar) *via* a carbocation containing in a six-membered ring,^90^ and (b) the reaction between phthalaldehyde and 2,7-dihydroxynaphthalene to give naphthopleiadene 1 (NP 1), *via* a carbocation contained within a seven-membered ring which cannot aromatise *via* loss of proton.

For the diverse applications listed above, the triptycene component generally requires a multi-step synthesis and laborious chromatographic purification of intermediates and product.^79,80^ Despite some newly developed methods,^81–85^ triptycene and its derivatives are still predominantly prepared by the Diels–Alder reaction between an anthracene derivative and a reactive benzyne intermediate.^86,87^ It should be noted that even unsubstituted triptycene is an expensive starting material, presumably due to the inherent difficulties and hazards in scaling-up reactions involving benzyne.^88,89^ Frustrated by the time-consuming preparation of triptycene monomers for making PIMs, we are developing methods for their rapid synthesis that can be readily performed on a large-scale. Here we report the simple one-step synthesis of a triptycene-like compound, which has a novel bridged bicyclic framework composed of a single benzene and two naphthalene units fused to two carbon bridging atoms, for which we propose the name naphthopleiadene (NP, Fig. 1). The readily prepared derivative NP 1 (Fig. 2), which contains in-built hydroxyl functionality, is useful for further synthetic elaboration as demonstrated by the synthesis of a very rigid cavitand, a microporous network polymer, a solution-processable PIM, and the unsubstituted NP hydrocarbon (Fig. 4).

## Results and discussion

Acid-mediated reactions between aldehydes and electron-rich aromatic compounds were considered for the synthesis of triptycene derivatives, similar to those used for the efficient assembly of resorcinarenes (ESI Fig. 1†).^91–93^ Naïvely, it was anticipated that these could be formed from the double addition of an appropriate aromatic compound to phthalaldehyde, however, literature reports of such reactions with benzene derivatives present no evidence of any triptycene product.^90,94–97^ Instead, it is proposed that 9-phenyl anthracenes are produced *via* a carbocation contained within a six-membered ring, from which the loss of a proton enables the efficient formation of the extended aromatic system (Fig. 2a). On consideration of this mechanism, it was reasoned that a triptycene-like product may be formed if the reaction pathway to 9-phenylanthracene could be blocked by avoiding the six-membered ring intermediate. For example, the double addition of 2,7-dihydroxynaphthalene to phthalaldehyde was anticipated to form a carbocation intermediate contained within a seven-membered ring, which would not be compatible with aromatization (Fig. 2b).

Using reaction conditions optimised for the synthesis of resorcinarenes (ESI Fig. 1†),^98,99^ heating an acidified ethanol solution of a 2 : 1 mole ratio of 2,7-dihydroxynaphthalene and phthalaldehyde rapidly produced a colourless solid. Intriguingly, ^1^H and ^13^C NMR analysis showed that a highly symmetric product was obtained in high purity. A single crystal X-ray diffraction study confirmed the formation of a novel bridged bicyclic framework with two sp^3^ hybridised bridging carbons, to which are fused a single benzene ring and two naphthalene units, the latter at their *peri*-(1,8)-positions. Like triptycene, the planes of the three fused aromatic units are fixed at an angle of ∼120° relative to each other (Fig. 2b). The related triptycene-like framework, consisting of two benzene rings and a single naphthalene unit, was reported in 1967.^100,101^ This bridged bicyclic compound was considered a derivative of the unsaturated hydrocarbon pleiadene,^102,103^ for which the seven-membered ring evoked the Pleiades of Greek mythology, therefore, it was named 7,12-(1′,2′-benzeno)-7,12-dihydropleiadene (Fig. 1).^100,101^ Using the same IUPAC endorsed nomenclature,^104^ we suggest the systematic name of 7,12-(1′,8′-naphthaleno)-7,12-dihydropleiadene for the novel molecular framework, and the trivial name naphthopleiadene (NP) for the parent hydrocarbon (Fig. 1), the synthesis of which is described below (Fig. 4).

Optimisation of the reaction conditions for the synthesis of 1,6,2′,7′-tetrahydroxynaphthopleiadene (NP 1) indicated that only a few drops of concentrated aqueous HCl (37%) are required and that simple alcohols perform best as solvent (ESI Table 1†). NP 1 was produced directly in good yield (∼70%, first crop) and required no further purification except for the removal of alcohol in a drying oven. Additional product (∼20%, second crop) can be obtained by removal of the solvent from the filtrate followed by recrystallisation. Remarkably, the maximum yield is produced within only a few minutes after heating to reflux. Alternatively, a similar yield and purity of product was obtained at ambient temperature, but the reaction took longer to complete (∼24 h). The low cost of the precursors and undemanding reaction conditions allows for large-scale synthesis with, for example, 70 g of NP 1 being obtained easily (ESI Table 1 and Fig. 2†). Water as reaction solvent gave a good crude yield but the resulting solid contained a purple impurity, which required recrystallisation from ethanol to remove, therefore, negating any potential benefits from its use.

**Fig. 3:**
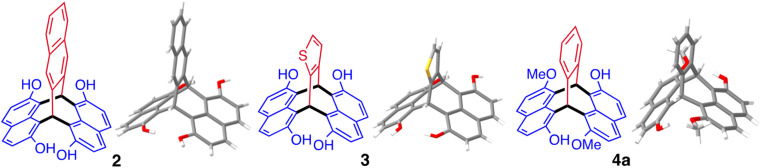
The structures derived from single crystal; XRD of triptycene-like compounds 2, 3 and 4a.

Replacement of phthalaldehyde in the reaction with 2,3-naphthalenedialdehyde or 1,2-thiophenedialdehyde also provided triptycene-like products 2 and 3, respectively (Fig. 3). Similarly, 2-hydroxy-7-methoxynapthalene reacts with phthalaldehyde to give a mixture of the isomeric products 1,7′-dihydroxy-6,2′-dimethoxy-naphthopleiadene (4a) and 1,2′-dihydroxy-6,7′-dimethoxy-naphthopleiadene (4b), which could be separated by using the relatively poor solubility of the former in acetone. The same reaction conditions using 2,7-dimethoxynaphthalene and phthalaldehyde failed to provide 1,6,2′,7′-tetramethoxynaphthopleiadene (5). However, by adapting an efficient preparation of methoxy-substituted triphenylmethanes,^105^ using BF_3_·OEt_2_ in dichloromethane, NP 5 was obtained in moderate yield with its structure also confirmed by single crystal XRD analysis.

**Fig. 4:**
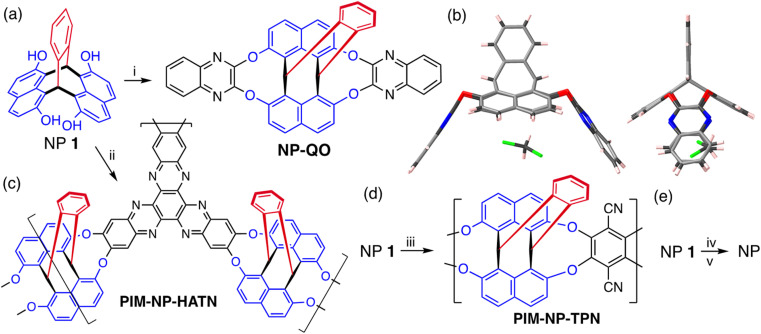
(a) The synthesis of cavitand NP-QO from NP 1: (i) 2,3-dichloroquinoxaline, K_2_CO_3_, DMF, 80 °C, 48 h, yield 100%. (b) Two views of the XRD structure of cavitand NP-QO showing the included CH_2_Cl_2_ molecule. (c) The synthesis of an ultramicroporous network polymer PIM-NP-HATN: (ii) 2,3,8,9,14,15-hexafluoro-5,6,11,12,17,18-hexaazatrinaphthylene, K_2_CO_3_, DMF, 120 °C, 48 h, yield 86%. (d) The synthesis of soluble PIM-NP-TPN: (iii) 2,3,5,6-tetrafluoroterephthalonitrile, K_2_CO_3_, DMF, 30–120 °C, 160 h, yield 84%. (e) (iv) Trifluoromethanesulfonic anhydride, pyridine, 25 °C, 16 h. (v) Pd(OAc)_2_, 1,3-bis(diphenylphosphino)propane, Et_3_SiH, DMF, 60 °C, 1 h.

The structure of NP 1, with a short distance between adjacent hydroxyl groups (O to O distance = 2.74 Å), suggested a similar reactivity to that of the resorcinarenes, for which Cram demonstrated deep cavitand formation using the efficient reaction with 2,3-dichloroquinoxaline (ESI Fig. 1†).^106^ Similarly, the reaction between NP 1 and 2,3-dichloroquinoxaline proceeds in very high yield *via* the formation of two nine-membered tribenzo-1,4-dioxonine rings (Fig. 4a). A single crystal XRD analysis of the product NP-QO shows a symmetrical cavitand-like structure that contains a single molecule of dichloromethane held within the cavity (Fig. 4b). Unlike Cram's deep cavitands, which exists in rapidly exchanging *vase* and *kite* conformers,^92,107^ the structure of NP-QO appears fully fixed by the NP framework. Indeed, simple molecular mechanical modelling suggest that the conformer produce by the inversion of a single tribenzo-1,4-dioxonine ring is ∼60 kJ mol^−1^ higher in energy due to the steric effect of the NP bridgehead hydrogen (ESI Fig. 3†).

The highly efficient formation of the tribenzo-1,4-dioxonine unit, demonstrated by the synthesis of cavitand NP-QO, suggested that polymers may also be prepared by similar aromatic nucleophilic substitution reactions. Hence, the synthesis of both a network and a non-network polymer was attempted by the reactions between NP 1 and 2,3,8,9,14,15-hexafluoro-5,6,11,12,17,18-hexaazatrinaphthylene^108^ or 2,3,5,6-tetrafluorophthalonitrile, respectively (Fig. 4c and d), with structural characterisation performed by solid-state NMR (ESI Fig. 4†). Thermal Gravimetric Analysis (TGA) of both polymers showed a mass loss of only ∼27% at 850 °C, providing very high char yields that are consistent with the predominately aromatic structure of the polymers (ESI Fig. 5†). For the resulting network polymer NP-HATN, there was low N_2_ adsorption at 77 K with the shape of the isotherm suggesting slow rate of uptake (ESI Fig. 6†). However, an impressive CO_2_ uptake of 3.3 mmol g^−1^ at 1 bar (273 K) suggests significant microporosity with the pore size distribution being predominantly ultramicroporous (<0.7 nm), which combined with high rigidity, severely reduces the rate of N_2_ adsorption (ESI Fig. 7†). Gas adsorption of the powdered form of the non-network polymer NP-TPN confirmed intrinsic microporosity with significant N_2_ uptake at 77 K allowing an apparent SA_BET_ of 635 m^2^ g^−1^ and total pore volume of 0.40 ml g^−1^ to be estimated. It may be that the non-network structure of PIM-NP-TPN allows some swelling during N_2_ adsorption, which facilitates greater total uptake. PIM-NP-TPN proved soluble in polar aprotic solvents, and once the polymerisation was optimised, a high average molecular mass (*M*_n_ = 140 000 g mol^−1^) was obtained as measured by gel permeation chromatography. Despite this, only brittle self-standing films of PIM-NP-TPN could be cast from DMF solution, which proved too fragile for gas permeability measurements. It should be noted that the ability to fabricate robust self-standing films from many PIMs, despite their fused ring structures, has been attributed to the relative flexibility of the dibenzodioxane linking group.^109^ However, a comparison of the flexibility of the dibenzodioxane linking group with that of tribenzo-1,4-dioxonine, using molecular mechanical modelling (ESI Fig. 8†), suggests that the latter is much more rigid, which is likely to be detrimental to the film forming properties of PIM-NP-TPN. Nevertheless, a solution-processable PIM, that contains cavitand-like structures, may be useful for making adsorbents or sensors and, hence, microporous polymers derived from NP 1 is the focus of continuing work.^110^

As suggested by a reviewer, the viability of using NP 1 as a precursor for making the previously unreported parent NP hydrocarbon (Fig. 1) was established by using the protodehalogenation of its pseudohalide triflate ester (Fig. 4e).^111^ Although optimisation of this reaction is required, the successful synthesis of NP was confirmed by ^1^H and ^13^C NMR and high-resolution mass spectroscopy. It was found that the chemical shift of the ^1^H NMR signal for the bridgehead hydrogens of the resulting NP (*δ*_H_ = 5.4 ppm, ESI Fig. 15†) is similar to those of triptycene and benzopleiadene.^101^ In comparison, the bridgehead hydrogens of NPs 1–5 are strongly deshielded relative to those of unsubstituted NP with values in the range 6.8–7.3 ppm, similar to those reported for triptycene derivatives that also contain hydroxyl or methoxy groups adjacent to the bridgehead.^50,112,113^ The bridgehead hydrogens of NP-QO have an extraordinary chemical shift of 8.79 ppm, which may be attributed to the electron-withdrawing nature of the quinoxaline unit combined with the effect of its ring current.

## Conclusions

With the increasing complexity of modern synthetic chemistry, it is gratifying to obtain a novel compound, which possesses both an interesting structure and useful reactivity, from a very simple reaction between two readily available precursors. The ease in which NP 1 is prepared suggests that it will have further applications beyond cavitand and microporous polymer synthesis. For example, NP 1 could replace various hydroxylated triptycene precursors, each of which require a complex multi-step synthesis,^114^ in the preparation of pincer catalysts^50^ and materials for which the structure and properties are controlled by triptycene self-assembly. Potential target materials include liquid crystals,^112^ highly ordered thin-films^113^ and polymers with unusual mechanical properties.^12,14,115^ As noted by Swager, triptycene self-assembly is driven by the requirement to eliminate intermolecular free volume (IMFV), thus favouring the interlocking of triptycene units.^17,18^ As the naphthalene units of NP generate larger IMFV than the benzene rings of triptycene (Fig. 1), the NP component may enhance self-assembly in these materials, in addition to being suitable for scale-up for commercial development. Finally, like their 2,7-dihydroxynaphthalene and 2,7-dimethoxynaphthalene precursors, solutions of NP 1 and NP 5 show visible blue fluorescence (ESI Fig. 16 and 17†), which may form the basis for applications in sensing.^116^

## Data availability

The data supporting this article have been included as part of the ESI.† Crystallographic data has been deposited at the CCDC with the following accession numbers: NP 1 (2286805); NP 2 (2286808); NP 3 (2286809); NP 4a (2286807); NP 5 (2286804) and NP-QO (2286806) and can be obtained from https://www.ccdc.cam.ac.uk/structures/.

## Author contributions

MKA led the synthetic effort. CY prepared PIM-NP-TPN, SP prepared NP 5. YL prepared NP 2 and its precursor. DT and SP prepared the NP parent hydrocarbon. DT obtained the UV/Visible and fluorescence spectra. GSN carried out single crystal XRD analysis, NBM conceived the project and wrote the first draft of the manuscript. All authors contributed to writing the final version of the manuscript.

## Conflicts of interest

There are no conflicts to declare.

## Supplementary Material

SC-OLF-D4SC02755H-s001

SC-OLF-D4SC02755H-s002
