# Supplementary material for: Triptycene-like naphthopleiadene as a readily accessible scaffold for supramolecular and materials chemistry
Source: Chem Sci. 2024 Aug 27;15(36):14968–76. doi: 10.1039/d4sc02755h (PMC11348350; doi:10.1039/d4sc02755h)
Supplement: SC-015-D4SC02755H-s001 [file SC-015-D4SC02755H-s001.pdf]

Supplementary Information (SI) for Chemical Science.
This journal is © The Royal Society of Chemistry 2024

Triptycene-like naphthopleiadene as a readily accessible scaffold for supramolecular and materials chemistry

Md Khairul Amin[1,2], Chunchun Ye[1], Shuhua Pang[1], Yuancheng Liu[1], Dominic Taylor[1], Gary S. Nichol[1], Neil B. McKeown[1]*

[1]EaStCHEM, School of Chemistry, University of Edinburgh, David Brewster Road, Edinburgh, EH9 3FJ, UK

[2]Chemistry Discipline, Khulna University, Khulna 9208, Bangladesh.

Synthesis. General Methods

Unless stated otherwise, all reagents were acquired from commercial sources and used without further purification. The reaction progress was monitored by thin layer chromatography (TLC). The melting points were recorded using a Stuart SMP10 digital melting point apparatus. FTIR spectra of the samples were recorded using a Shimadzu IR Affinity-1S FTIR spectrophotometer in the range of 4000-400 cm^{-1}. The ^{1}H and ^{13}C NMR spectra of the synthesized compounds were acquired in a suitable deuterated solvent using an Avance Bruker AVA 500 or PRO 500 instrument at 298 K. The chemical shifts (d) are recorded in parts per million (ppm). The multiplicity is noted as singlet (s), doublet (d), doubled-doublet (dd), multiplet (m), or broad (br). High-resolution mass spectrometry (HRMS) was performed by using a Thermo Finnigan MAT900XP Sector or Bruker UltrafleXtreme MALDI TOF/TOF instrument utilizing electron impact ionization (EI) or matrix assisted laser desorption/ionization (MALDI). Single crystal X-ray structures were analysed by Bruker D8 Venture diffractometer, Rigaku Oxford diffraction SuperNova diffractometer, or Rigaku Oxford diffraction XCalibur diffractometer equipped with an Oxford Cryosystems 800 low-temperature device operating at 100 K or 120 K temperature. To measure the surface area and pore size of the polymers, low-temperature (77 K) N_2 and CO_2 (273 K) adsorption/desorption isotherms were recorded using a Quantachrome Quadrasorb Evo instrument. Accurately weighed polymeric materials (~100 mg) were degassed overnight under high vacuum at 110 °C prior to analysis. Gel permeation chromatography (GPC) analyses were carried out using dimethylformamide solution (~1.0 mg ml^{-1}) using an Agilent 1100 instrument equipped with a RI detector, operating at a flow rate of 1.0 ml min^{-1}. Calibration was achieved using a polymethylmethacrylate (PMMA) standard. Thermo-gravimetric analysis (TGA) of the materials was carried out using a NETZSCH STA 449F1 instrument with a heating rate of 10 °C min^{-1} up to 850 °C under nitrogen atmosphere.

Synthesis of naphthopleiadene NP 1

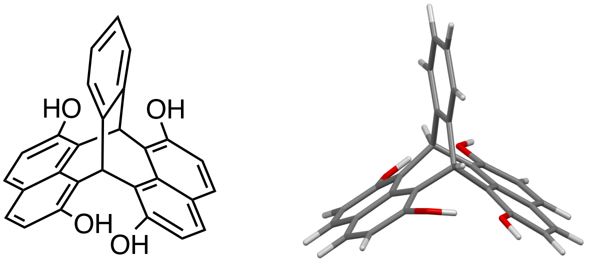

A solution of 2,7-dihydroxynaphthalene (1.19 g, 7.46 mmol), phthalaldehyde (0.50 g, 3.73 mmol), and 37% hydrochloric acid (0.1 ml) in methanol (15 ml) was heated at reflux for 4 h to afford naphthalopleiadene **1** as white solid, which was isolated by filtration and washed with cold ethanol (1.15 g, 74%). Mp: > 300 °C; FTIR (ν, cm^{-1}): 3198, 2980, 2867, 1611, 1511; ^{1}H NMR (500 MHz, DMSO) δ 9.97 (s, 4H), 7.49 (d, J = 8.7 Hz, 4H), 7.40 (dd, J = 6.6, 3.6 Hz, 2H), 7.16 (dd, J = 6.6, 3.6 Hz, 2H), 6.98 (d, J = 8.7 Hz, 4H), 6.84 (s, 2H); ^{13}C NMR (126 MHz, DMSO) δ 152.0, 141.9, 133.9, 129.1, 128.5, 127.6, 125.3, 120.4, 115.6, 48.5; HRMS (ESI, *m/z*): $[M+H]^+$ calculated for $C_{28}H_{19}O_4$: 419.1278, found: 419.1269; X-ray Crystallography: crystal size 0.37 × 0.24 × 0.15 mm, triclinic, space group *P*-1(2), *a* = 10.2376(7), *b* = 10.4837(6), *c* = 12.0766(7) Å, α = 107.559(3)°, β = 103.934(3)°, γ = 103.550(3)°, *V* = 1131.50(13) $Å^3$, *Z* = 2, *T* = 100.0 K, *R* = 3.28% (1 molecule of isopropanol included in crystal). Deposition number CCDC 2286805.

Table 1. Variation of reaction conditions relative to general procedure above. All yields quoted are from isolated products of high purity (from NMR) except where indicated.

Solvent	Time (h)	Temp	Yield (%, 1st crop)	Yield (%, 2nd crop)	Yield (% Total)
Methanol	4 h	Reflux	74	NM[a]	NM
Methanol	4 h	Reflux	69[b]	NM	NM
Ethanol	17 h	Reflux	67	22	89
Ethanol	4 h	Reflux	70	22	92
Ethanol	2 h	Reflux	71	NM	NM
Ethanol	1 h	Reflux	74	NM	NM
Ethanol	0.5 h	Reflux	73	NM	NM
Ethanol	0.1 h	Reflux	70	NM	NM
Ethanol	5 h	rt	35	NM	NM
Ethanol	23 h	rt	70	NM	NM
Ethanol	1 h (no aq. HCl)	Reflux	0	0	0
Isopropanol	4 h	Reflux	65	26	91
Water	4 h	Reflux	83 (impure)	NM	70[c]

[a]NM = not measured; [b]large-scale reaction (70 g product); [c]after recrystallisation of crude

Synthesis of benzonaphthopleiadene NP 2

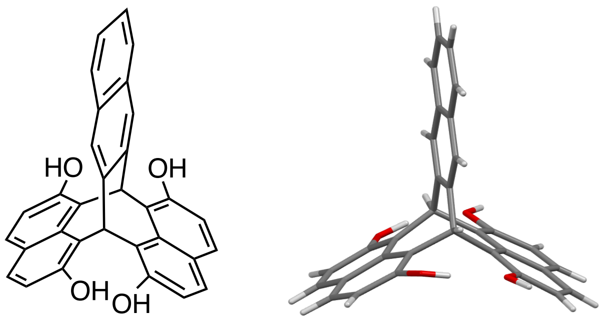

A solution of 2,7-dihydroxynaphthalene (3.48 g, 21.72 mmol), naphthalenedialdehyde (2.0 g, 10.86 mmol), and 37% hydrochloric acid (0.3 ml) in methanol (25 ml) was heated at reflux for 4 h to afford **2** as a white solid (3.61 g, crude yield 71%). Mp: > 300 °C; FTIR (ν, cm^{-1}): 3662, 3172, 2864, 1616, 1440; ^{1}H NMR (500 MHz, DMSO) δ 9.96 (s, 4H), 7.93 (s, 2H), 7.86 (dd, J = 6.3, 3.3 Hz, 2H), 7.47 (d, J = 8.7 Hz, 4H), 7.40 (dd, J = 6.3, 3.3 Hz, 2H), 7.09 (s, 2H), 6.98 (d, J = 8.7 Hz, 4H);^{13}C NMR (126 MHz, DMSO) δ 152.1, 140.9, 134.0, 133.0, 129.0, 127.5, 126.2, 126.0, 125.2, 120.8, 115.6, 48.5; HRMS (EI, *m/z*): [$M+H^+$] calculated for $C_{32}H_{21}O_4$: 469.1434, found: 469.1425; X-ray Crystallography: crystal size 0.18 × 0.17 × 0.06 mm, triclinic, space group *P*-1(2), a = 10.4986(5), b = 11.6083(6), c = 12.2425(6) Å, α = 97.683(4)°, β = 106.716(4)°, γ = 100.894(4)°, V = 1374.87(12) $Å^3$, Z = 2, T = 120.01(10) K, R = 5.98%. Deposition number CCDC 2286808.

Synthesis of naphthothiophenopleiadene NP 3

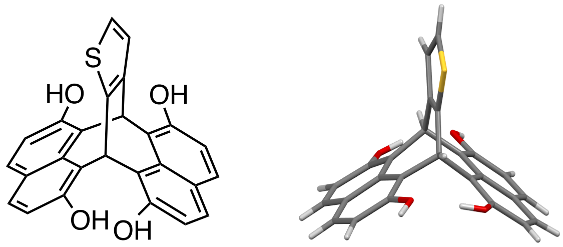

A solution of 2,7-dihydroxynaphthalene (2.06 g, 12.84 mmol), thiophenedialdehyde (0.90 g, 6.42 mmol), 37% hydrochloric acid (0.1 ml) in methanol (20 mL) was heated at reflux for 4 h to afford the product **3** as an off-white solid (2.10 g, crude yield 77%). Mp: > 300 °C; FTIR (v, cm^{-1}): 3662, 3172, 2864, 1616, 1440; ^{1}H NMR (500 MHz, DMSO) δ 9.96 (s, 2H), 9.91 (s, 2H), 7.44-7.50 (m, 4H), 7.04 (d, J = 7.0 Hz, 2H), 7.03 (s, 1H), 6.93-6.98 (m, 4H), 6.91 (s, 1H), 6.88 (d, J = 7.0 Hz, 1H); HRMS (EI, *m/z*): [$M+H^+$] calculated for $C_{26}H_{17}O_4$: 425.0848, found: 425.0831; X-ray Crystallography: crystal size 0.35 × 0.26 × 0.07 mm, monoclinic, space group $P2_1/n$(14), a = 10.5199(2), b = 18.4144(3), c = 13.8648(3) Å, β = 98.763(2)°, α = γ = 90°, V = 2654.50(9) $Å^3$, Z = 4, T = 120.01(10) K, R = 5.72% (note two molecules of DMSO included in structure and that thiophene ring shows positional disorder in crystal). Deposition number CCDC 2286809.

Synthesis of naphthopleiadenes NP 4a and 4b

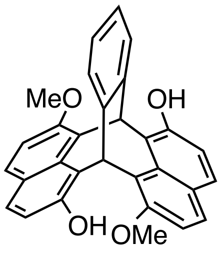

4a

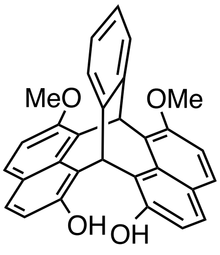

4b

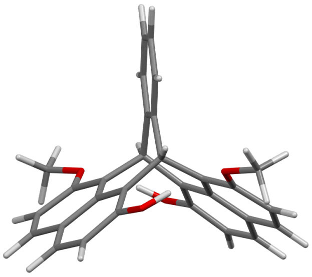

A solution of 7-methoxy-2-naphthol (2.60 g, 14.91 mmol), phthalaldehyde (1.00 g, 7.46 mmol), and 37% hydrochloric acid (0.2 ml) in methanol (20 ml) was heated at reflux for 4 h to afford a solid mixture of **4a** and **4b** isomers (2.37 g, 71%). Trituration using excess acetone isolated NP **4a** as a white solid (0.67 g, 20%). The solvent from the filtrate was evaporated and the white precipitate filtered and dried to yield NP **4b** (0.30 g, 9%).

4a Mp: > 300 °C; FTIR (*v*, cm^{-1}): 3410, 2949, 2845, 1614, 1457, 1242; ^{1}H NMR (500 MHz, DMSO) δ 9.56 (s, 2H), 7.56 (d, J = 8.8 Hz, 2H), 7.48 (d, J = 8.8 Hz, 2H), 7.33 (dd, J = 5.6, 3.4 Hz, 2H), 7.16 (d, J = 8.8 Hz, 2H), 7.12 (dd, J = 5.6, 3.4 Hz, 2H), 7.07 (s, 2H), 6.98 (d, J = 8.8 Hz, 2H), 4.01 (s, 6H); ^{13}C NMR (126 MHz, DMSO) δ 154.2, 153.0, 142.4, 134.1, 128.7, 128.6, 128.2, 127.3, 125.3, 123.8, 120.0, 116.4, 111.8, 58.0; HRMS (MALDI-TOP, *m/z*): [M^{+}] calculated for $C_{30}H_{22}O_{4}$: 446.15126, found: 446.15034; X-ray Crystallography: crystal size 0.25 × 0.15 × 0.06 mm, monoclinic, space group *C*2/*c*(15), a = 17.9101(6), b = 8.2287(3), c = 14.3468(5) Å, α = 107.559(3)°, β = 105.751(4)°, $\alpha = \gamma$ = 90°, V = 2034.98(12) $Å^{3}$, Z = 4, T = 100.01(10) K, R = 3.31%. Deposition number CCDC 2286807.

4b Mp: > 300 °C (decom.); FTIR (*v*, cm^{-1}): 3016, 2966, 2937, 1616, 1458, 1244; ^{1}H NMR (500 MHz, DMSO) δ 7.83 – 7.75 (m, 4H), 7.71 (dd, J = 10.6, 8.8 Hz, 4H), 7.49 (dd, J = 5.5, 3.2 Hz, 2H), 7.17 (s, 2H), 7.12 – 7.06 (m, 1H), 6.89 (d, J = 8.8 Hz, 2H), 4.04 (s, 6H); ^{13}C NMR (126 MHz, DMSO) δ 159.2, 155.6, 137.2, 135.4, 132.0, 130.4, 129.8, 128.2, 124.8, 118.8, 116.2, 111.2, 103.5, 77.6, 55.9; HRMS (MALDI-TOP, *m/z*): $[M-H]^{+}$ calculated for $C_{30}H_{21}O_{4}$: 445.14342, found: 445.14208.

Synthesis of naphthopleiadene NP 5

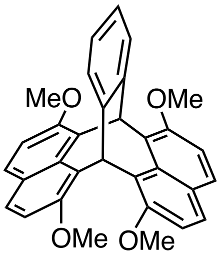

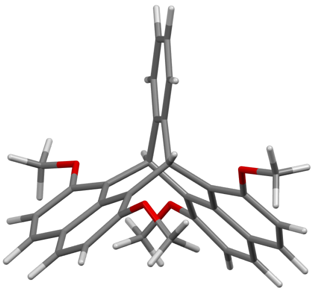

To a solution of 2,7-dimethoxynaphthalene (1.40 g, 7.46 mmol) and phthalaldehyde (0.50 g, 3.73 mmol) in dichloromethane (7 ml), boron trifluoride diethyl etherate (1.06 g, 7.46 mmol) was carefully added and the mixture was stirred at room temperature for 1 h. The reaction mixture was filtered, washed with dichloromethane, and recrystallized from dimethyl sulfoxide provide NP **5** as an off-white solid (1.01 g, 57%). Mp: > 300 °C; FTIR (*v*, cm^{-1}): 2968, 2864, 1612, 1510, 1247; ^{1}H NMR (500 MHz, $CDCl_3$) δ 7.55 (d, *J* = 8.9 Hz, 4H), 7.47 (dd, *J* = 5.5, 3.6 Hz, 2H), 7.27 (s, 2H), 7.14 (dd, *J* = 3.6, 5.5 Hz, 2H), 7.12 (d, *J* = 8.9 Hz, 4H), 4.06 (s, 12H);^{13}C NMR (126 MHz, DMSO) δ 154.6, 142.1, 133.4, 129.1, 128.3, 127.5, 125.7, 123.0, 112.2, 57.5; HRMS (ESI, m/z): $[M+H]^+$ calculated for $C_{32}H_{27}O_4$: 475.1904, found: 475.1891; X-ray Crystallography: crystal size 0.36 × 0.07 × 0.03 mm, orthorhombic, space group *Fdd2*(43), *a* = 30.9566(5), *b* = 17.6585(2), *c* = 8.2176(1) Å, *α* = *β* = *γ* = 90°, *V* = 4492.13(10) $Å^3$, *Z* = 8, *T* = 120.00(10) K, *R* = 2.82%. Deposition number CCDC 2286804.

Synthesis of cavitand NP-QO

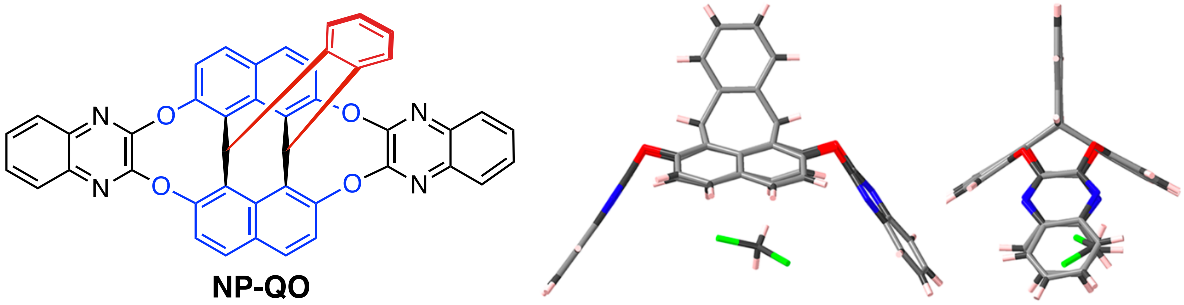

NP-QO

NP **1** (2.00 g, 4.78 mmol), 2,3-dichloroquinoxaline (2.09 g, 10.51 mmol), and anhydrous potassium carbonate (5.28 g, 38.24 mmol) in dry dimethylformamide (25 ml) were heated at 80 °C for 48 h to yield the **NP-QO** as a white solid (3.20 g, 100%). Mp: > 300 °C; FTIR (*v*, cm^{-1}): 2976, 2863, 1611, 1506, 917; ^{1}H NMR (500 MHz, $CDCl_3$) δ 8.79 (s, 2H), 8.09 (d, *J* = 8.9 Hz, 3H), 8.04 (dd, *J* = 6.0, 3.5 Hz, 5H), 7.85 (dd, *J* = 6.0, 3.5 Hz, 2H), 7.71 (dd, *J* = 6.4, 3.4 Hz, 4H), 7.59 (d, *J* = 8.9 Hz, 4H), 7.37 (dd, *J* = 6.4, 3.4 Hz, 2H); ^{13}C NMR (126 MHz, $CDCl_3$) δ 153.4, 151.6, 140.8, 139.9, 132.1, 131.7, 131.3, 129.8, 129.8, 129.7, 128.3, 128.2, 122.5, 39.6; HRMS (MALDI-TOP, *m/z*): $[M]^+$ calculated for $C_{44}H_{22}N_4O_4$: 671.17138, found: 671.17129; X-ray Crystallography: crystal size 0.46 × 0.28 × 0.19 mm, triclinic, space group *P*-1(2), *a* = 10.0081(3), *b* = 14.3562(5), *c* = 14.6779(4) Å, *α* = 63.466(3)°, *β* = 75.953(2)°, *γ* = 81.918(2)°, *V* = 1829.09(11) $Å^3$, *Z* = 2, *T* = 120.00(10) K, *R* = 4.86% (crystallised with 2 molecules of DCM). Deposition number CCDC 2286806.

Synthesis of Network microporous PIM-NP-HATN

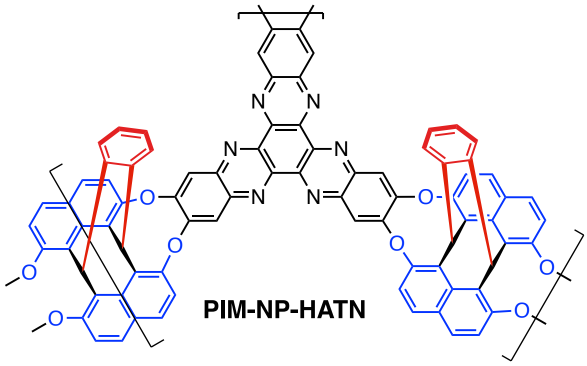

To a stirred solution of 2,3,8,9,14,15-hexafluoro-5,6,11,12,17,18-hexaazatrinaphthylene (1.50 g, 3.05 mmol) and NP **1** (1.91 g, 4.57 mmol) in dry dimethylformamide (110 ml) was added finely ground anhydrous potassium carbonate (5.05 g, 36.56 mmol). The reaction mixture was then heated at 120 °C for 48 h under nitrogen. On cooling, the reaction mixture was added to water (400 ml) and the solid product was collected by filtration and washed with deionized water, acetone, and methanol. Purification was achieved by heating the solid in hot dimethylacetamide, tetrahydrofuran, acetone, and methanol. The resulting orange-red powder was dried in a vacuum oven at 140 °C for 48 h (2.54 g, 86%), FTIR (v, cm^{-1}): 3294, 1612, 1200, 1082; ^{13}C NMR (solid state, 76 MHz) δ 152.6, 141.6, 127.7, 119.5, 38.9; SA_{BET} = 354 $m^2\ g^{-1}$, total pore volume = 0.13 $cm^3\ g^{-1}$ (p/p_0 = 0.98), CO_2 uptake = 73 $cm^3\ g^{-1}$ (273 K, 1 bar); TGA: 200 °C (onset decomposition temperature, 27% mass loss at 850 °C).

Synthesis of PIM-NP-TPN

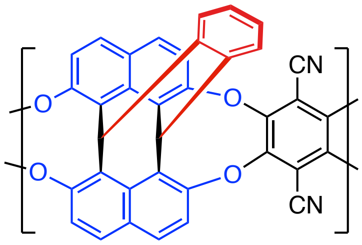

PIM-NP-TPN

NP **1** (10.0 g, 23.90 mmol), tetrafluoroterephthalonitrile (4.78 g, 23.90 mmol), anhydrous potassium carbonate (26.42 g, 191.18 mmol), and dry dimethylformamide (300 ml) were combined in a reactor to afford PIM-NP-TPN as a yellow solid (10.76 g, 84%). FTIR (*v*, cm^{-1}): 3039, 2232, 995; ^1H NMR (500 MHz, DMSO) δ 6.8-7.9 single very broad peak observed; ^{13}C NMR (solid state, 76 MHz) δ 152.8, 139.3, 127.8, 120.1, 39.1; GPC (DMF, vs. PMMA standards): M_n = 140,000 g mol^{-1}, M_w = 760,000 g mol^{-1}, M_w/M_n = 5.3; SA_{BET} = 635 $m^2\ g^{-1}$, total pore volume = 0.40 $cm^3\ g^{-1}$ (p/p_0 = 0.98), CO_2 uptake = 61 $cm^3\ g^{-1}$ (273 K, *1 bar*); TGA: 485 °C (onset decomposition temperature, 27% mass loss at 850 °C).

Synthesis of NP

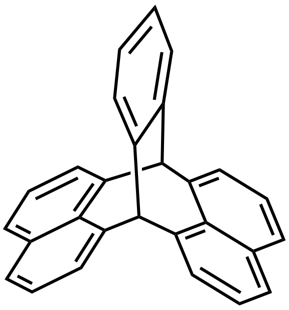

Trifluoromethanesulfonic anhydride (2.1 mL, 12 mmol) was added dropwise over a 10 min period to a stirred solution of NP **1** (1 g, 2.4 mmol) in dry pyridine (10 mL) at 0 °C under N_2. The mixture was then warmed to room temperature and stirred for 16 h. The reaction mixture was dropped into 50 mL water and the precipitate was collected via a filtration. After washing the solid with cold methanol and drying in oven at 100 °C overnight, the triflate ester intermediate was recrystallised from hot ethanol to yield as white solid (220 mg, 10%). MP: 268-270 °C, ^{1}H NMR (500 MHz, $CDCl_3$): δ_H (ppm) = 7.79 (d, J = 9.1 Hz, 4H), 7.73 (dd, J = 6.6, 3.5 Hz, 2H), 7.53 (d, J = 9.1 Hz, 4H), 7.36 (dd, J = 6.6, 3.5 Hz, 2H), 6.99 (s, 2H); ^{13}C NMR (126 MHz, $CDCl_3$): δ_C (ppm) = 145.6, 137.0, 132.9, 132.4, 130.7, 129.5, 129.3, 129.3, 120.7, 119.9, 117.3, 41.9; ^{19}F NMR (471 MHz, $CDCl_3$): δ_F (ppm) = -73.1 (s, 12F); HRMS (EI, m/z): $[M + Na]^+$ calculated for $C_{32}H_{14}F_{12}O_{12}S_4Na$: 968.9068, found: 968.9050. To a stirred solution of the crude triflate ester (0.5 g, 0.53 mmol), $Pd(OAc)_2$ (0.047 g, 0.21 mmol) and 1,3-bis(diphenylphosphino)propane (0.088 g, 0.21 mmol) in dry DMF (12 mL) at 60 °C was added Et_3SiH (0.85 mL, 5.5 mmol) and the reaction mixture was stirred for 1 hour. After cooling to room temperature, the reaction was quenched with water (60 mL) and the precipitate was collected by filtration and washed with MeOH to give the crude product as a dark grey solid. The crude was passed through a column of silica gel using an eluent of toluene and hexane (1:3) to give product **NP** as white solid (70 mg, 37%), which was purified further by sublimation. MP: >300 °C ^{1}H NMR (500 MHz, $CDCl_3$): δ_H (ppm) = 7.7-7.6 (m, 8H), 7.52 (dd, J = 5.5, 3.4 Hz, 2H), 7.35 (t, J = 7.1 Hz, 4H), 7.19 (dd, J = 5.6, 3.4 Hz, 2H), 5.68 (s, 2H). ^{13}C NMR (126 MHz, $CDCl_3$): δ_C (ppm) = 140.5, 139.7, 136.0, 129.5, 128.6, 128.1, 127.5, 127.1, 125.8, 64.8. HRMS (EI, *m/z*): $[M-H]^+$ calculated for $C_{28}H_{19}$: 355.14813, found: 355.1476.

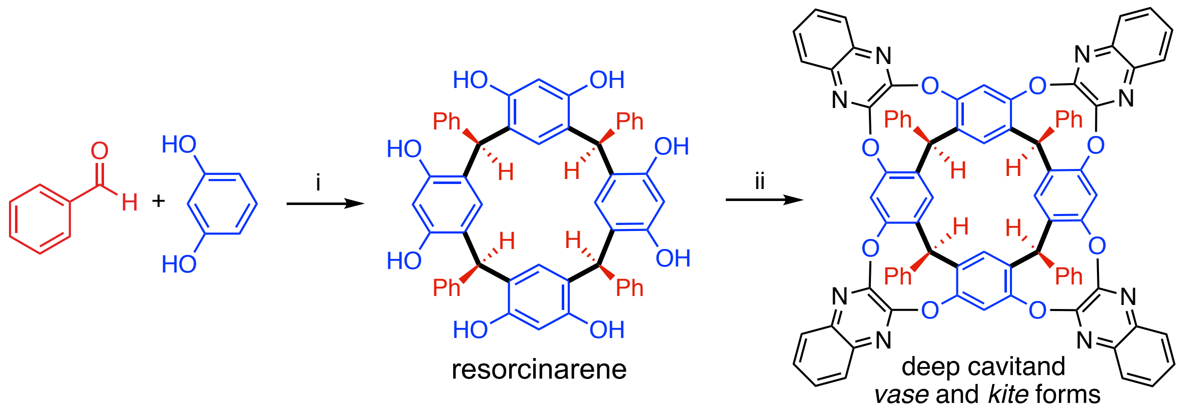

SI Fig. 1. Synthesis of tetraphenyl resorcinarene and the deep cavitand derived from it. *Reagents and conditions.* i. aq. HCl, ethanol reflux. ii. 2,3-dichloroquinoxaline, K_2CO_3, DMF, 80 °C, 48 h.

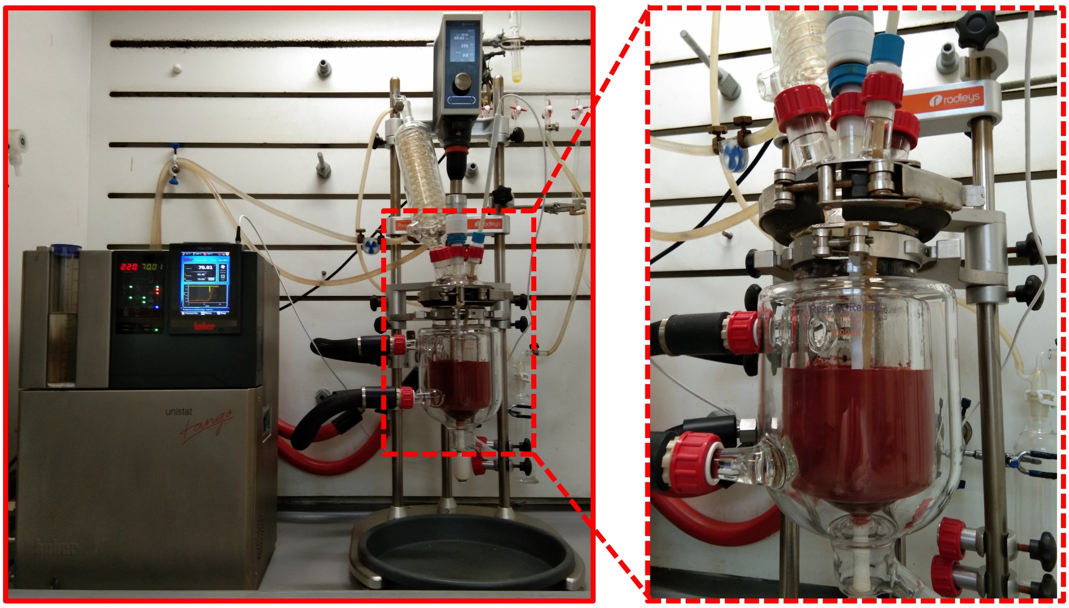

SI Fig. 2. Large-scale (70 g) synthesis of NP **1**.

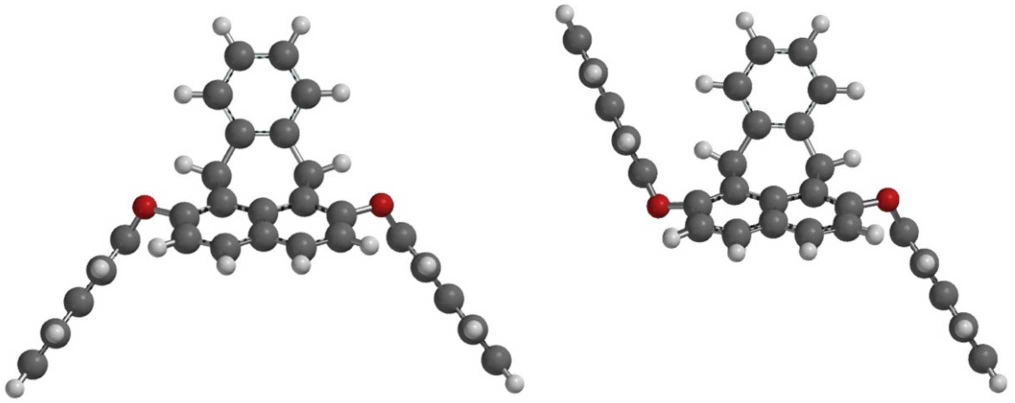

SI Fig. 3. Analysis of two potential conformers of cavitand **NP-QO** caused by the inversion of the tribenzo-1,4-dioxonine ring fused to a rigid NP unit, from its lowest energy position (left) using molecular mechanical calculations (Spartan). The inverted structure (right) is higher in energy by 60 kJ mol^{-1} due to the steric effect of the bridgehead hydrogen. Note there is also very a high energy cost associated with conformational movement of fused benzene within the tribenzo-1,4-dioxonine ring (SI Fig. 8).

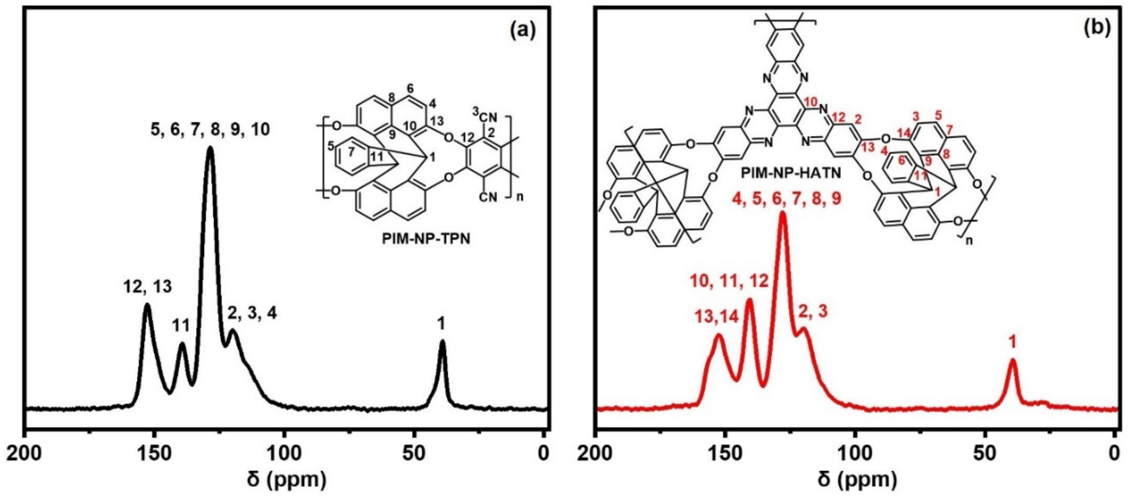

SI Fig. 4. Solid state ^{13}C NMR spectra of **PIM-NP-TPN** (black) and **PIM-NP-HATN** network (red) polymer with suggested peak assignments.

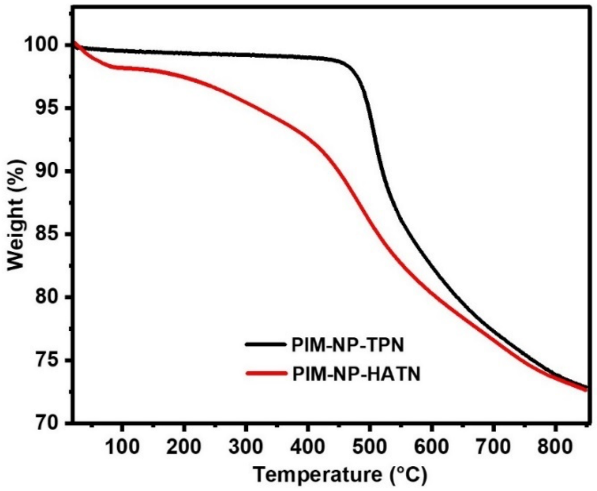

SI Fig. 5. Thermogravimetic analysis of **PIM-NP-TPN** (black) and **PIM-NP-HATN** network (red). Both polymers have shown high char yield at 850 °C.

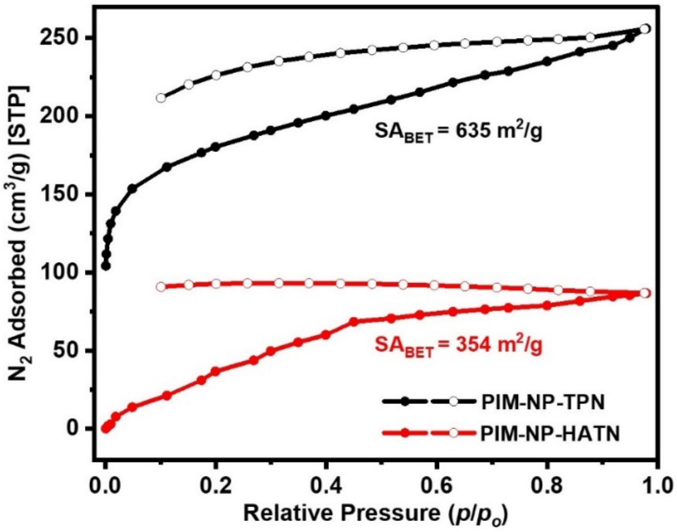

SI Fig. 6. Nitrogen adsorption isotherms acquired at 77 K for **PIM-NP-TPN** (black) and **PIM-NP-HATN** network (red).

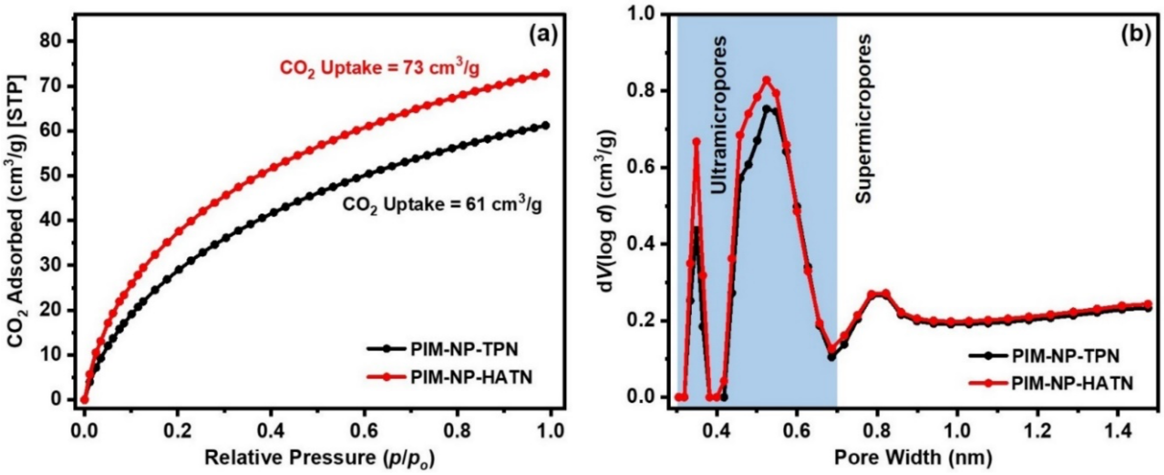

SI Fig. 7. (a) Carbon dioxide adsorption isotherms of **PIM-NP-TPN** (black) and **PIM-NP-HATN** network polymer (red) at 273 K and (b) their pore size distribution (PSD) curves calculated from carbon dioxide adsorption data using non-local density functional theory (NLDFT) model.

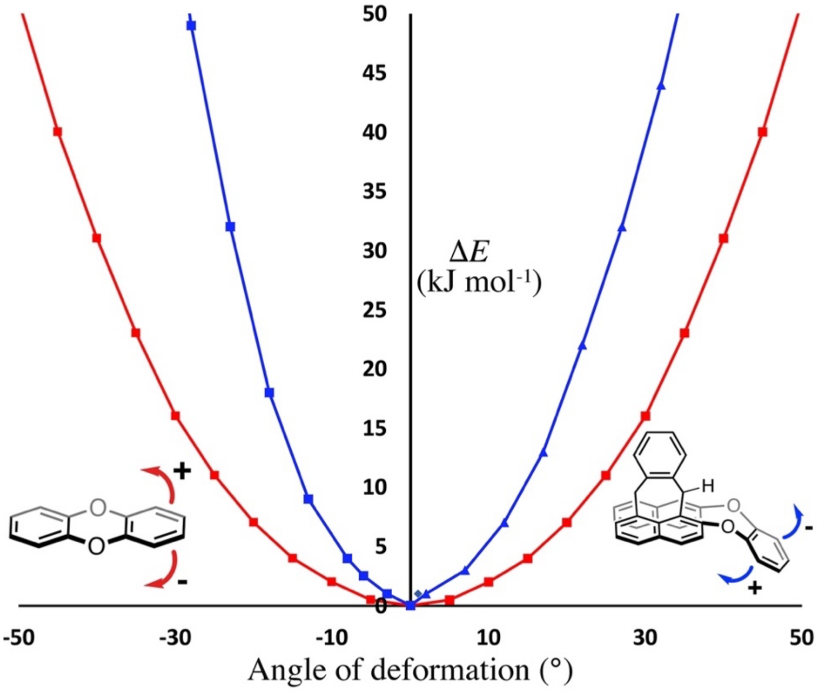

SI Fig. 8. Comparison of the energy cost required to move the benzene ring of a dibenzodioxin (red) or tribenzo-1,4-dioxonine, when fused to a rigid NP unit, (blue) from its lowest energy position. These molecular mechanical calculations (Spartan), suggest that the latter is more rigid than the former, which may contribute to the brittle nature of PIMs made using the formation of tribenzo-1,4-dioxonine link

NMR characterisation of NP compounds.

^{1}H NMR (500 MHz, DMSO) δ 9.97 (s, 4H), 7.49 (d, *J* = 8.7 Hz, 4H), 7.40 (dd, *J* = 7.5, 3.8 Hz, 2H), 7.16 (dd, *J* = 5.6, 3.2 Hz, 2H), 6.98 (d, *J* = 8.7 Hz, 4H), 6.84 (s, 2H).

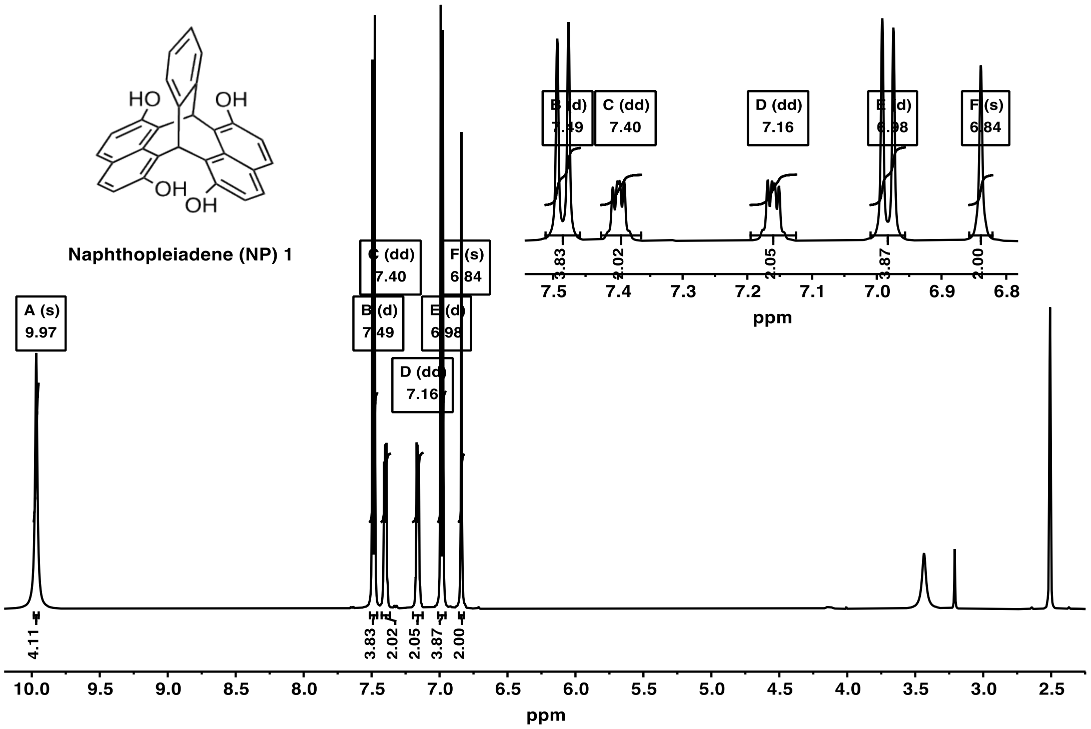

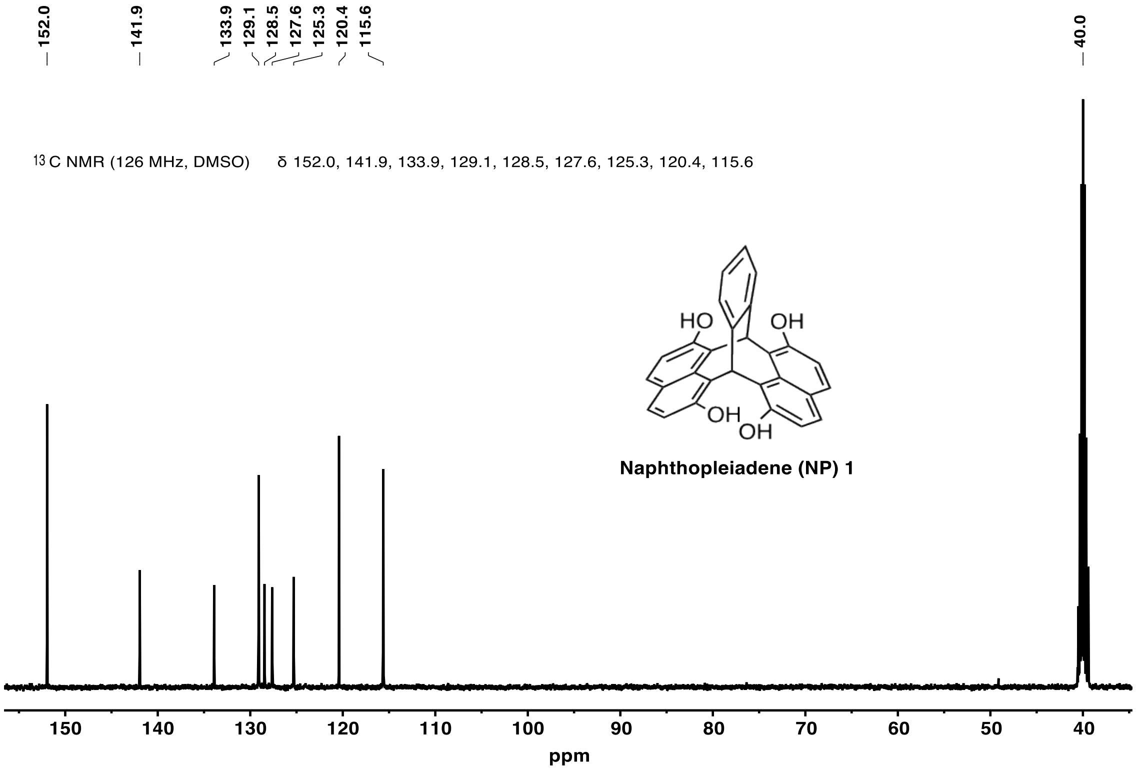

SI Fig. 9. ^{1}H NMR (top) and ^{13}C NMR (bottom) spectra of naphthopleiadene **1**.

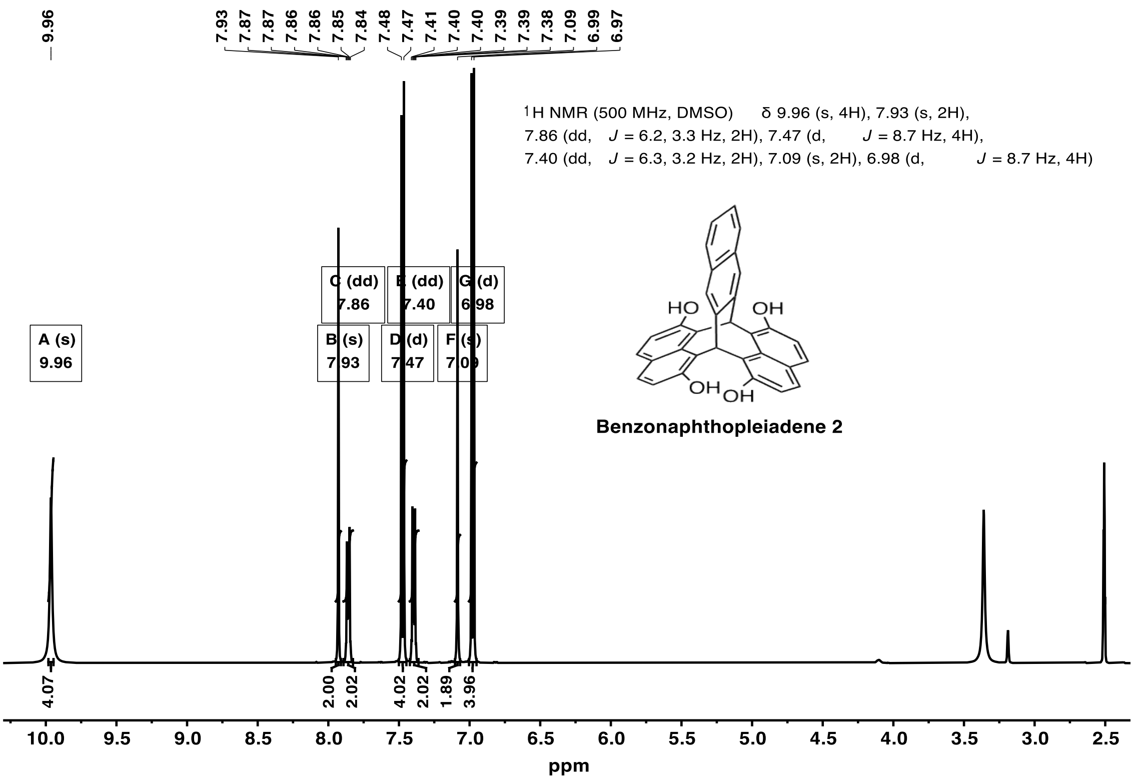

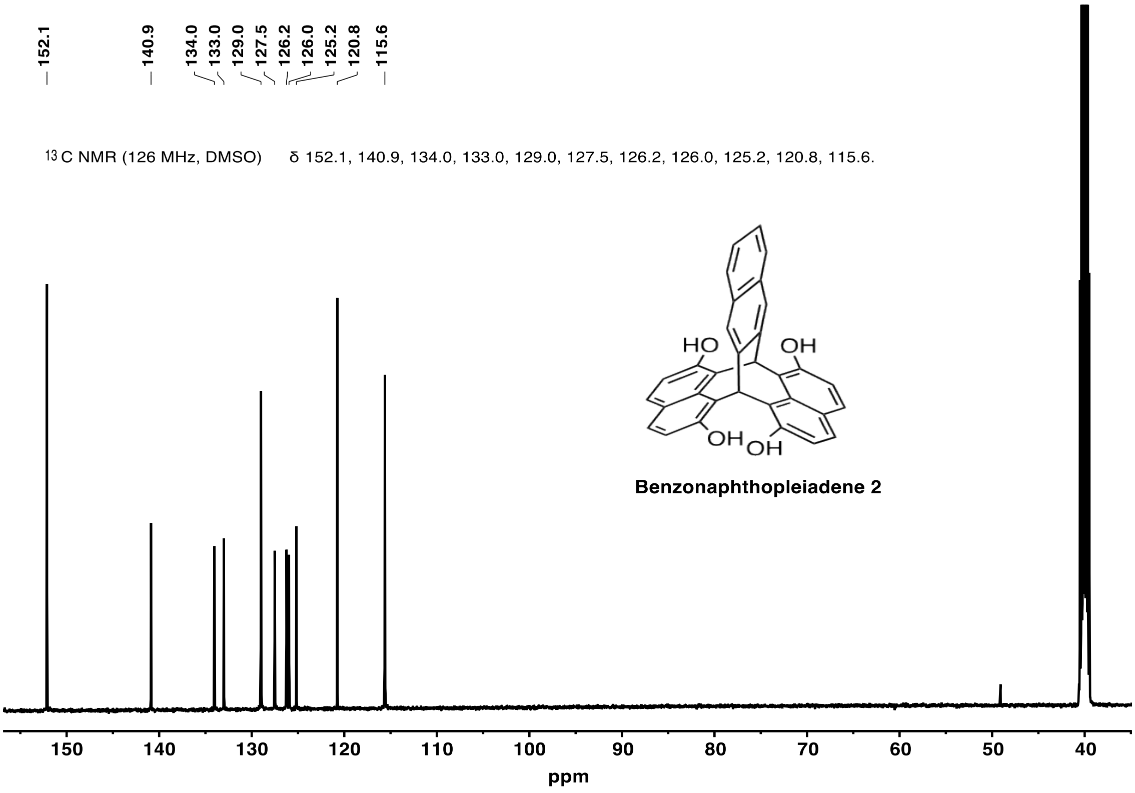

SI Fig. 10. ^{1}H NMR (top) and ^{13}C NMR (bottom) spectra of benzonaphthopleiadene **2**.

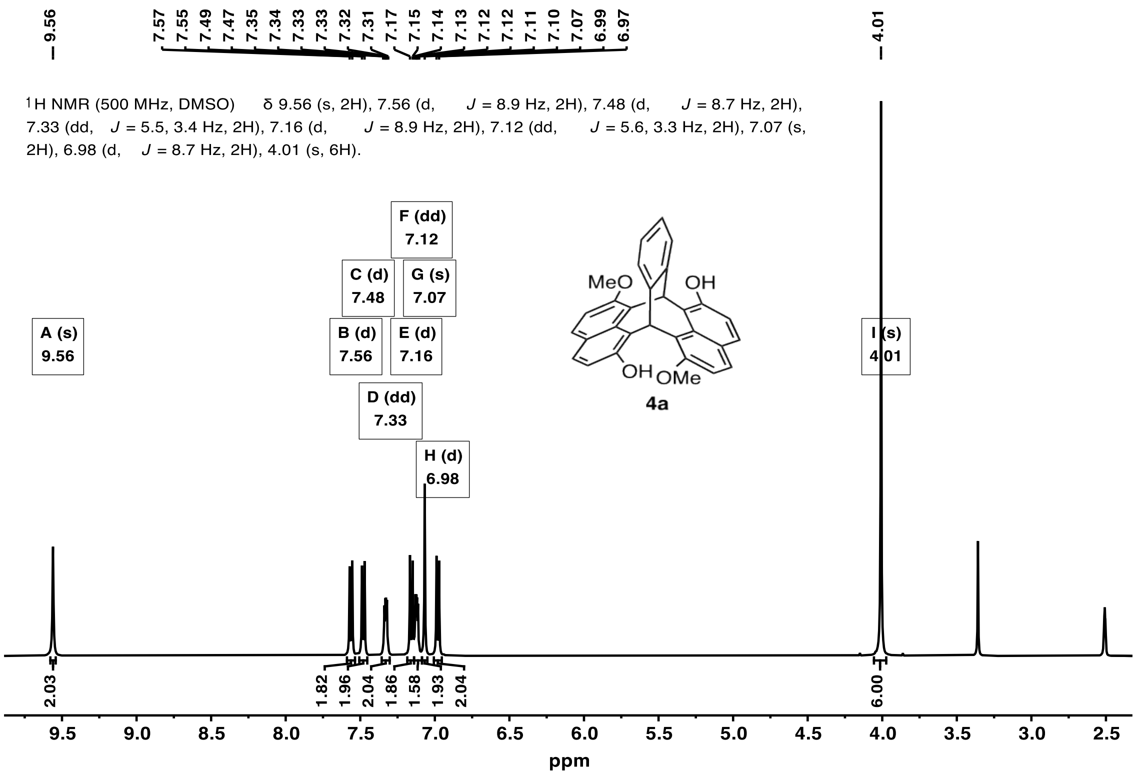

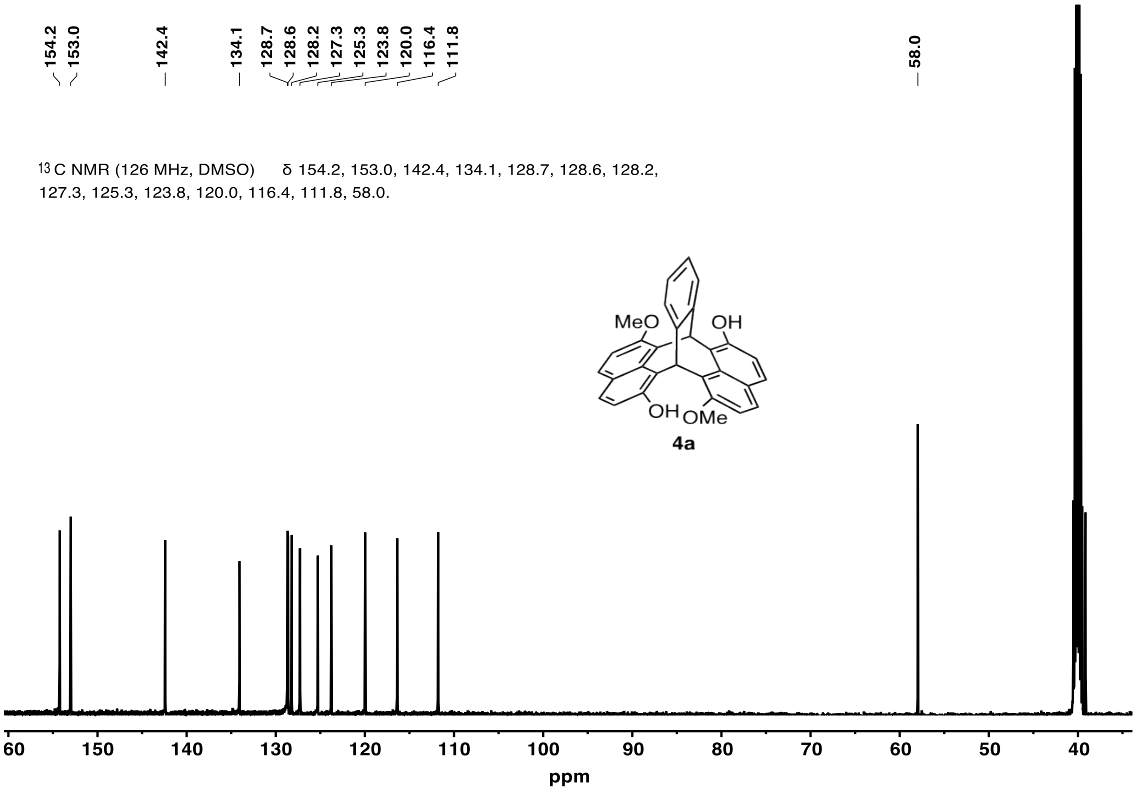

SI Fig. 11. ^{1}H NMR (top) and ^{13}C NMR (bottom) spectra of naphthopleiadenes **4a**.

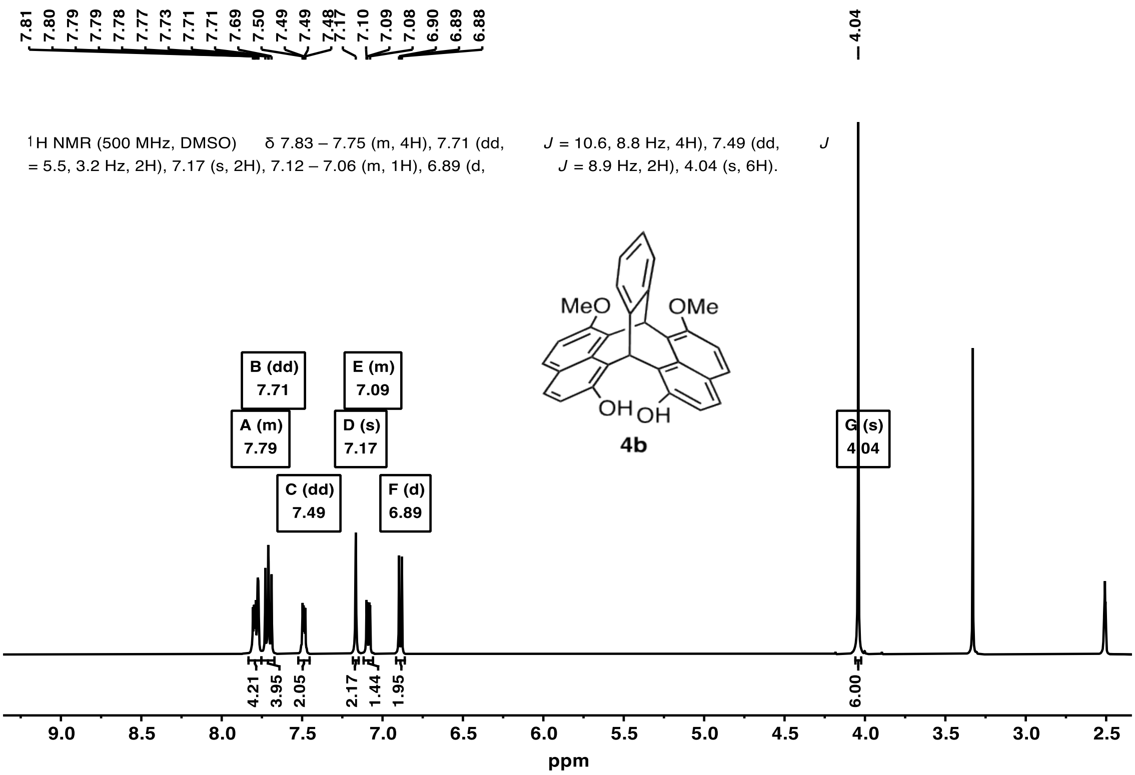

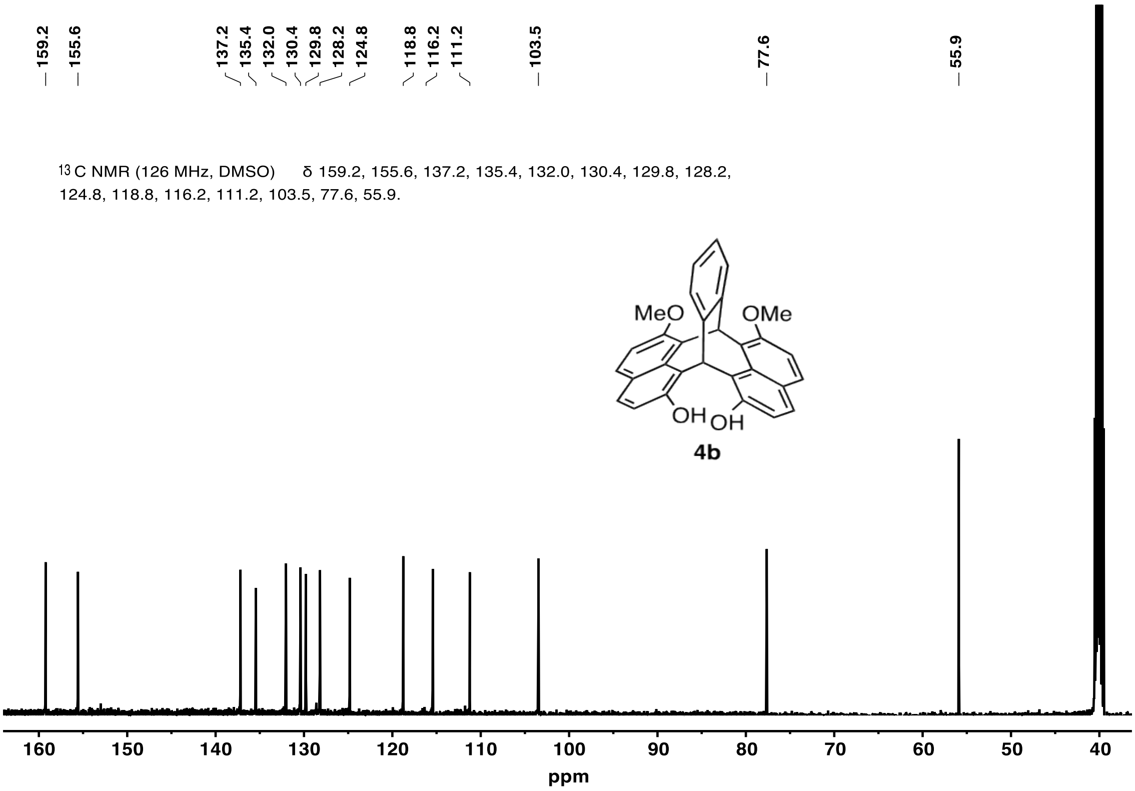

SI Fig. 12. ^{1}H NMR (top) and ^{13}C NMR (bottom) spectra of naphthopleiadenes **4b**.

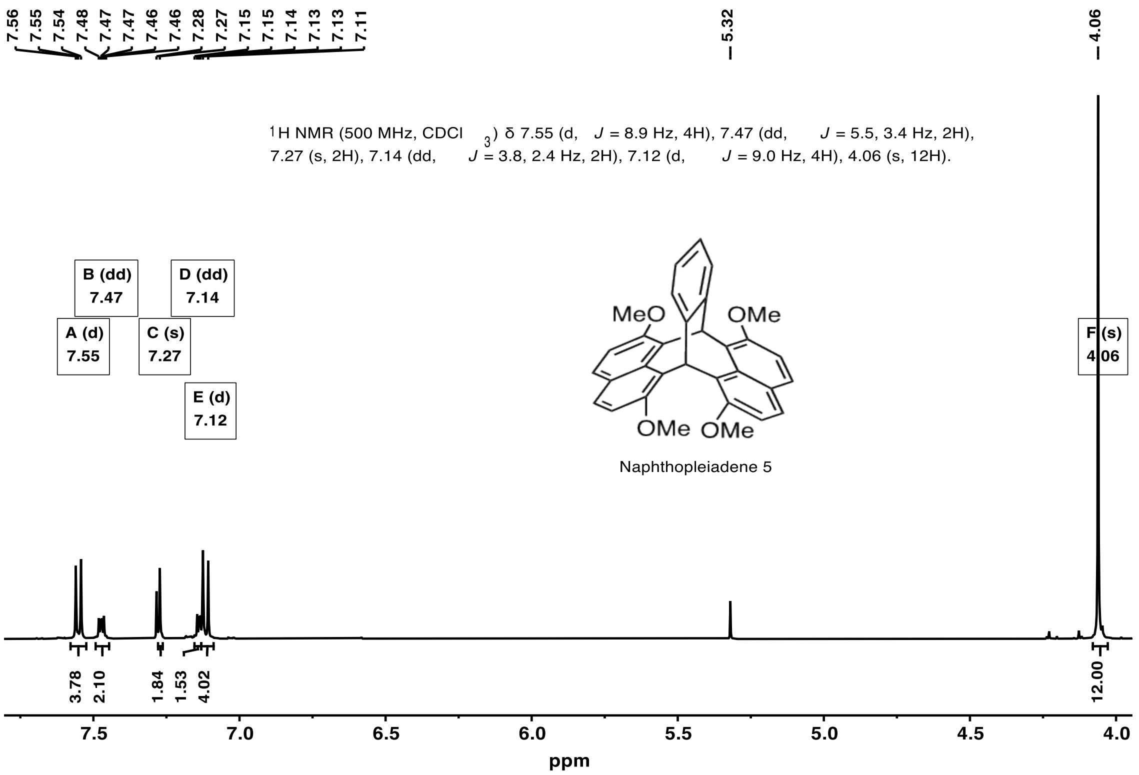

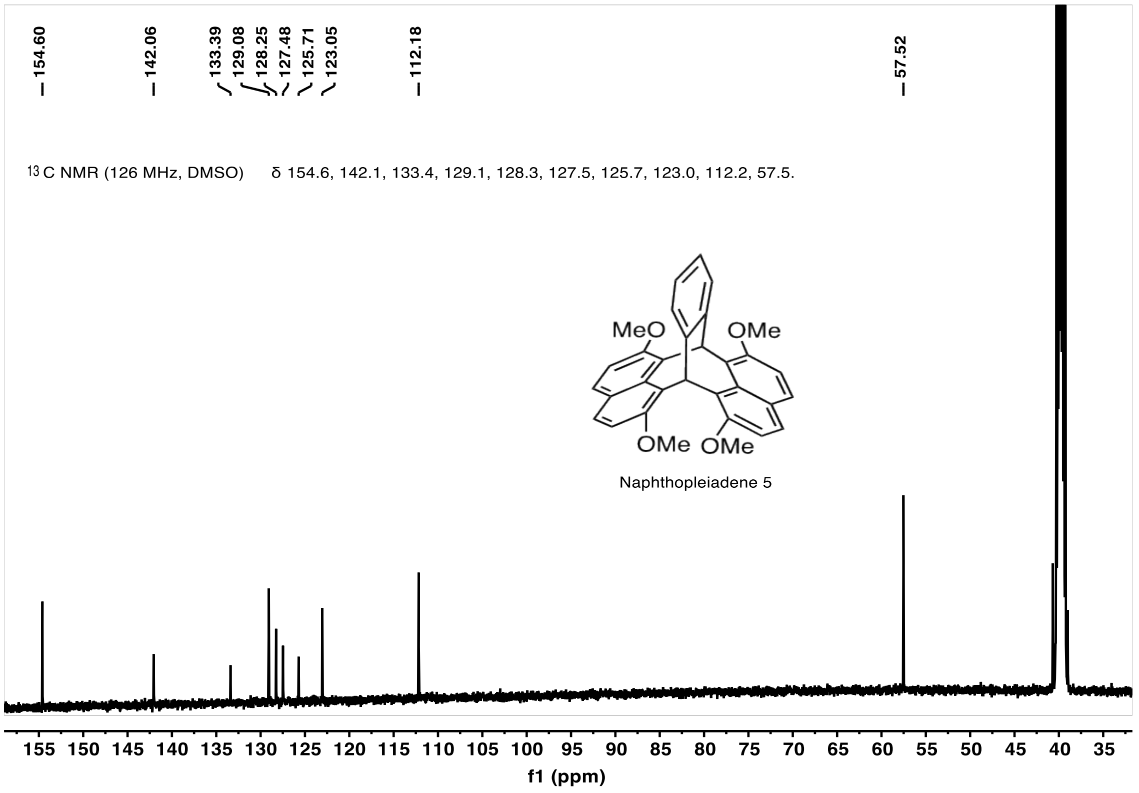

SI Fig. 13. ^{1}H NMR (top) and ^{13}C NMR (bottom) spectra of naphthopleiadene **5**.

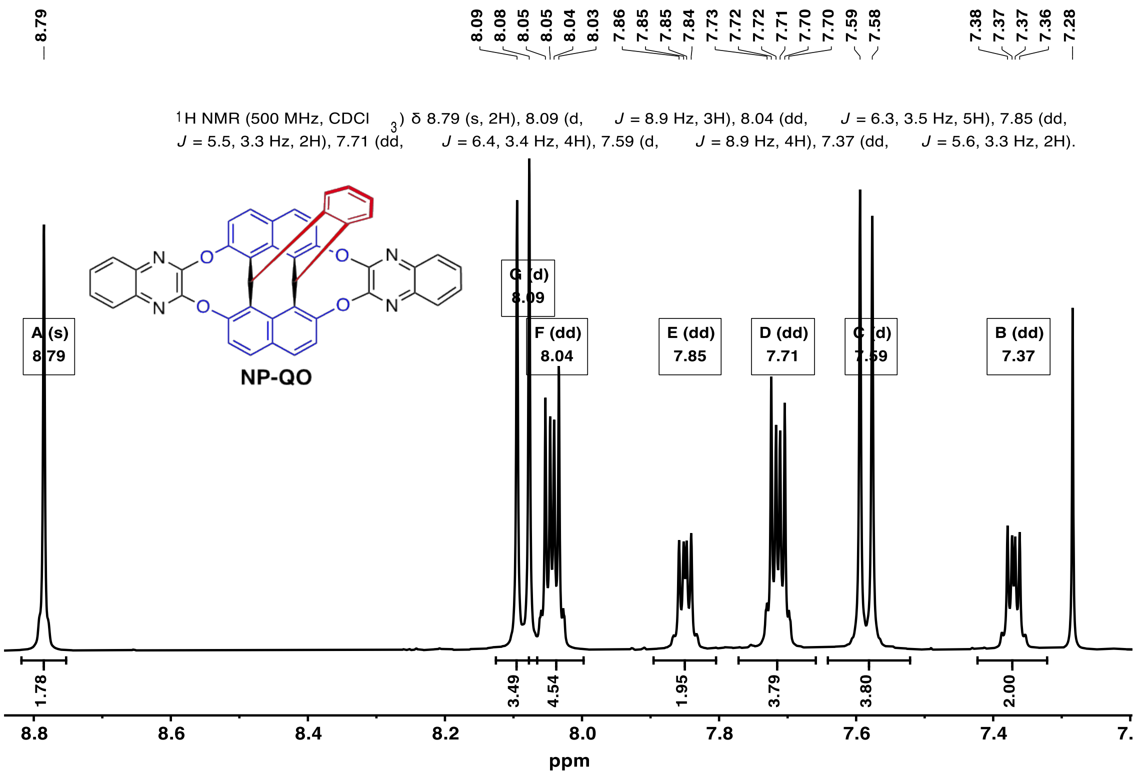

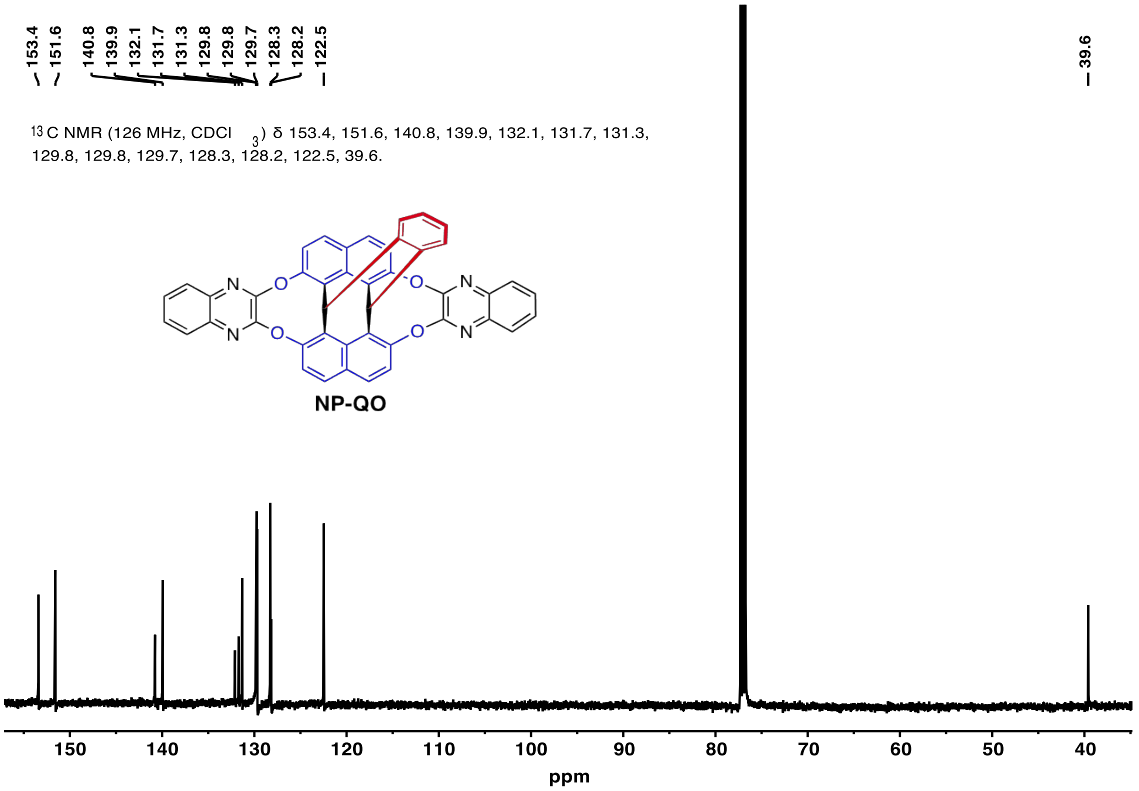

SI Fig. 14. ^{1}H NMR (top) and ^{13}C NMR (bottom) spectra of cavitand **NP-QO**.

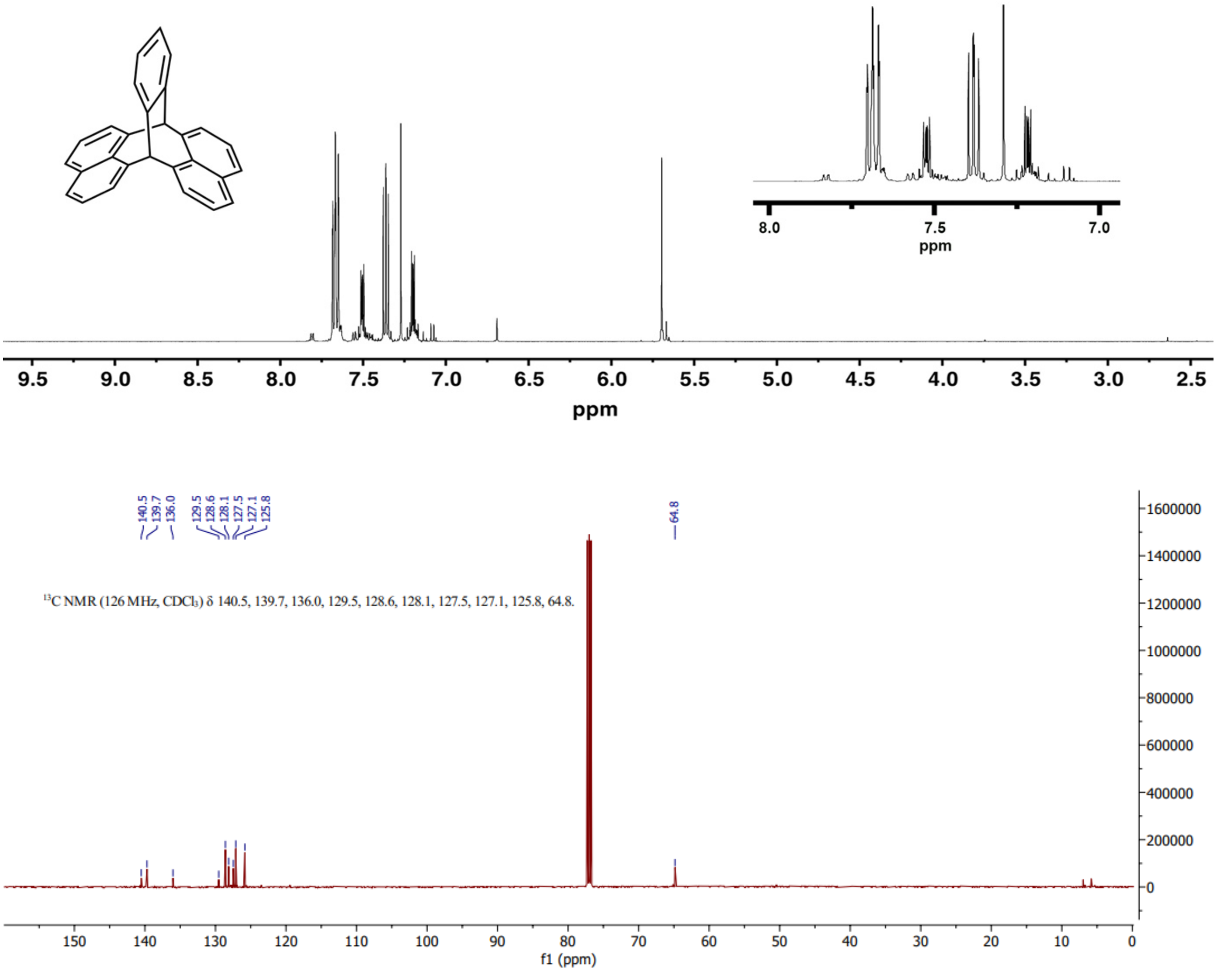

SI Fig. 15. ^{1}H NMR (top) and ^{13}C NMR (bottom) spectra of NP in $CDCl_3$.

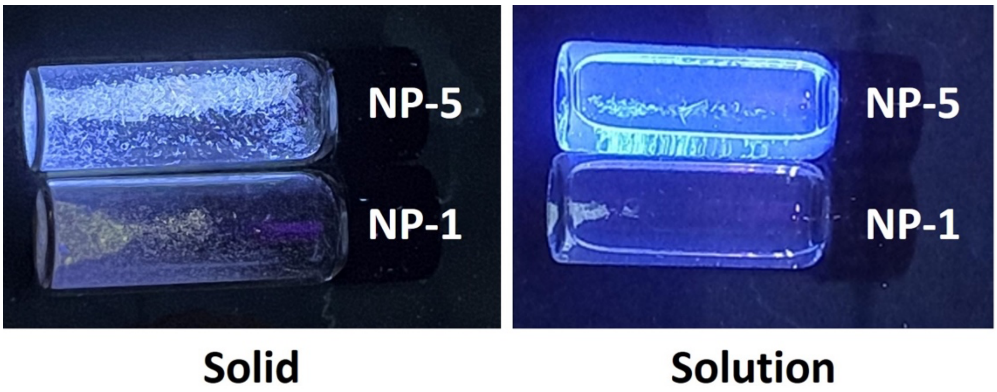

SI Fig. 16. Solution and solid-state fluorescence of NP **1** and NP **5**.

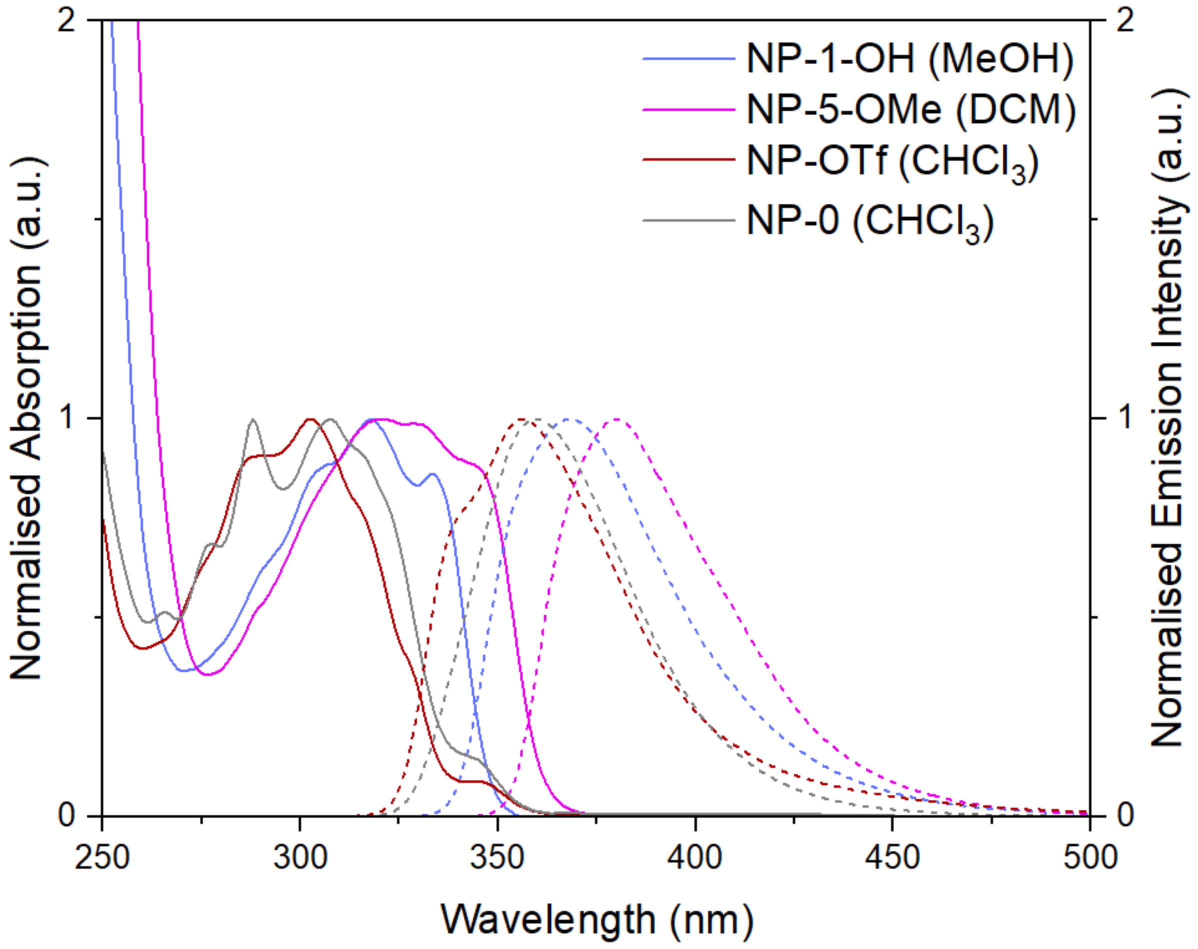

SI Fig. 17. UV/visible adsorption and emission spectra of NP **1** in methanol (blue,), NP **5** in DCM (pink), the triflate ester of NP **1** in $CHCl_3$ (brown,) and NP in $CHCl_3$ (grey). For the emission spectrum the excitation was at 320 nm.
